# The phosphorylation of sorting nexin 5 at serine 226 regulates retrograde transport and macropinocytosis

**DOI:** 10.1371/journal.pone.0207205

**Published:** 2018-11-12

**Authors:** Nao Itai, Tsukasa Shimazu, Takayuki Kimura, Issei Ibe, Ryo Yamashita, Yasushi Kaburagi, Taeko Dohi, Takashi Tonozuka, Toshifumi Takao, Atsushi Nishikawa

**Affiliations:** 1 Division of Applied Biological Chemistry, United graduate School of Agricultural Science, Tokyo University of Agriculture and Technology, 3-5-8 Saiwai-cho, Fuchu, Tokyo, Japan; 2 Department of Applied Biological Science, Graduate School of Agriculture, Tokyo University of Agriculture and Technology, 3-5-8 Saiwai-cho, Fuchu, Tokyo, Japan; 3 Department of Diabetic Complications, Diabetes Research Center, Research Institute, National Center for Global Health and Medicine, 1-21-1 Toyama, Shinjuku-ku, Tokyo, Japan; 4 Department of Gastroenterology, Research Center for Hepatitis and Immunology, Research Institute, National Center for Global Health and Medicine, 1-7-1 Kohnodai, Ichikawa, Chiba, Japan; 5 Laboratory of Protein Profiling and Functional Proteomics, Institute for Protein Research, Osaka University, 3–2 Yamadaoka, Suita, Osaka, Japan; Institut Curie, FRANCE

## Abstract

Sorting nexin 5 (SNX5), a member of sorting nexin family, plays an important role in membrane trafficking, including the retrograde trafficking of the cation independent mannose 6-phosphate receptor (CI-M6PR) and macropinocytosis. Using ESI-LCMS/MS analysis, we confirmed that SNX5 serine 226 is phosphorylated. Since SNX5 forms heterodimers with SNX1 or SNX2, we examined the effect of phosphorylation at S226 on the heterodimer formations. Wild-type and mutants of SNX5, in which S226 was mutated to a glutamic acid or an alanine, were expressed in 8505C cells. In pull-down assays using SNX5 as bait, only the S226E mutant failed to precipitate both SNX1 and SNX2. Confocal microscopy data indicated that the wild type and S226A mutant were colocalized with SNX1 and SNX2 in endosomes, but the S226E was not. SNX5 and SNX6 support each other's functions and are involved with CI-M6PR retrograde trafficking. In SNX5 and SNX6 double knockdown cells, CI-M6PR was dispersed and colocalized with the endosomal marker EEA1. In a rescue experiment using SNX5 mutants, the S226A rescued CI-M6PR localization, similar to control cells, but S226E did not. Furthermore, the decrease in the uptake of dextran by macropinocytosis in SNX5 knockdown cells was recovered by the expression of rescue-wild type or S226A mutant, but not by the rescue-S226E mutant. These observations indicate that SNX5 constitutive phosphorylation that mimics the mutant S226E decreases the active SNX5 in these cells. The phosphorylation of SNX5 regulates the dimerization with SNX1 or SNX2, and this suggests that it controls membrane trafficking and protein sorting.

## Introduction

In mammalian cells, endocytic pathways function to maintain cellular physiological function and occasionally play a role in combating infections by variant pathogens [1–2]. Functional aberrations can sometimes cause disorders, including the development of neurodegenerative diseases and cancer [3–5]. Recent studies have revealed that the pathways are subject to elaborate regulation by post-translational modification (PTM) such as protein phosphorylation and ubiquitination [6–7].

Macropinocytosis arises from the actin-mediated membrane ruffling of the plasma membrane in response to growth factors such as epidermal growth factor (EGF), platelet-derived growth factor (PDGF) and macrophage colony stimulating factor (CSF-1), resulting in the bulk endocytosis of solute molecules, antigens and nutrients [2]. Macropinosome formation is phosphoinositide 3-kinase (PI3K) dependent [8] and its subsequent maturation involves the progressive recruitment of late endosomal and lysosomal components [9]. It has been reported that some SNX-BAR proteins are involved in macropinocytosis. [10–11].

The retrograde transport, endosome to the trans-Golgi network (TGN), is essential for the recycling of transmembrane proteins such as cation independent mannose 6-phosphate receptor (CI-M6PR). The transmembrane proteins are packaged in the vesicles which were budded from the endosome and then sorted to TGN [12]. It has been reported that some of SNX-BAR proteins promote budding and tubulation of endosome membrane [13].

SNX-bin, Amphiphysin, and Rvs (SNX-BAR) proteins belong to the sorting nexin (SNX) family, which participate in diverse types of intracellular transport [14–16]. They contain a phox homology (PX) domain that recognizes the intracellular membrane through binding to selected phosphatidylinositols (PtdIns) and the BAR domain that remodels the membrane and dimerizes with other SNX-BAR proteins [14–17]. The heterodimer of SNX1 or SNX2 and SNX5 or SNX6 which belong to the SNX-BAR subfamily are considered to be involved in the retromer pathways which are retrograde transport from endosome to TGN [18–21] and recycling pathway to plasma membrane [22–24]. Furthermore, it has been reported to be linked to membrane remodeling and tubulation of endosome [13].

We previously reported that cell spreading, in the human colon cancer cell line Colo201, was induced by treatment with the broad-spectrum kinase inhibitor staurosporine [25]. During this morphological change in the cells, we found that the isoelectric point of SNX5 had changed. In studies to elucidate the basis for this change, we identified three novel phosphorylation sites on SNX5. As SNX5 has been shown to have an important role in retrograde transport and macropinocytosis, we asked whether phosphorylation of these sites influences the function of SNX5. If so, this would represent the first demonstration of the mechanism responsible for regulating a membrane trafficking system by SNX5. We report herein on an investigation of the role played by the phosphorylation of SNX5 in these processes.

## Materials and methods

### Antibodies and regents

The antibodies used were purchased commercially from the following manufacturers: mouse monoclonal anti-FLAG (SIGMA-ALDRICH), rabbit polyclonal anti-FLAG (SIG-MA-ALDRICH), mouse monoclonal anti-SNX1 (sc-136247; Santa Cruz), mouse mono-clonal anti-SNX2 (sc-136072; Santa Cruz), rabbit polyclonal anti-SNX5 (Santa Cruz), rabbit polyclonal anti-SNX6 (Santa Cruz), rabbit polyclonal anti-GAPDH (Santa Cruz), mouse monoclonal anti-EEA1 (MBL), mouse monoclonal anti-TGN46 (SIGMA-ALDRICH), ECL^TM^ anti-mouse IgG, horseradish peroxidase linked whole antibody (GE Healthcare), ECL^TM^ anti-rabbit IgG, horseradish peroxidase linked whole antibody (GE Healthcare), Alexa Fluor 488 goat anti-rabbit IgG (Invitrogen), Alexa Fluor 568 goat anti-mouse IgG (Invitrogen). Anti-CI-M6PR rabbit polyclonal was kindly provided by Dr. Jack Rohrer (Zurich University of Applied Sciences). Human recombinant EGF and FITC-conjugated dextran 70 k were obtained from Wako pure chemicals industries and Molecular probe, respectively.

### Plasmid and adenovirus preparation

SNX5 cDNA, purchased from Invitrogen, was subcloned into C-Terminal p3XFLAG-CMV-14 plasmid (Sigma-Aldrich). Site-directed mutagenesis was performed according to the manufacture’s protocol using a QuikChange Site-Directed Mutagenesis Kit (Agilent Technologies, Santa Clara). The PCR primers that were used to introduce the SNX5 mutants as follows, 5’-TTTAAGAAGGCTGTGGCCGCTCATGAAGTC-3’ and 5’-GACTTCATGAGCGGCCACAGCCTTCTTAAA-3’; T139A/S141A/S142A (TSS-AAA), 5’-GCTGTGTTTAAGAAGGAGGTGGAGGAGCATGAAGTCTTTCTT-3’ and 5’-AAGAAAGACTTCATGCTCCTCCACCTCCTTCTTAAACACAGC-3’; T139E/S141E/S142E (TSS-EEE), 5’-TCAGCGGCTTGCCGCTCACCCTGTTCTCGCTAAAGATCGC-3’ and 5’-GCGATCTTTAGCGAGAACAGGGTGAGCGGCAAGCCGCTGA-3’; S151A/S152A/S157A (SSS-AAA), 5’-CTTTCTTCAGCGGCTCGAGGAGCACCCTGTTCTCGAGAAAGATCGCAACTTT-3’ and 5’- AAAGTTGCGATCTTTCTCGAGAACAGGGTGCTCCTCGAGCCGCTGAAGAAAG-3’; S151E/S152E/S157E (SSS-EEE), 5’-ATAGGATCAAAGATGCATGTGTGAAAGCTG-3’ and 5’- CAGCTTTCACACATGCATCTTTGATCCTAT-3’; S226A, and 5’- TAGGATCAAA-GATGAGTGTGTGAAAGCTGA-3’ and 5’- TCAGCTTTCACACACTCATCTTTGATCCTA-3’; S226E, 5’- TAGGATCAAAGATGATTGTGTGAAAGCTGA-3’ and 5’- TCAGCTTTCACACAATCATCTTTGATCCTA-3’; S226D, 5’- TAGGATCAAAGA-TATCTGTGTGAAAGCTGA-3’ and 5’-TCAGCTTTCACACAGATATCTTTGATCCTA-3’; S226I, respectively.

Adenoviruses bearing wild type or mutant SNX5 were prepared using the ViraPower Adenovirus Expression System (Invitrogen) according to the manufacturer's instructions. Briefly, the SNX5 3xFLAG cDNAs were subcloned into the pENTR 1A vector. After purification of the plasmids, the cDNA inserts were transferred to the pAd/CMV/V5-DEST vector by means of the Gateway system using LR clonase. After digesting the linearized plasmids with PacI, they were transfected into HEK293A cells using lipofectamine LTX (Invitrogen). The resulting 293A cells were then cultured for 8 days in Dulbecco’s modified Eagle’s medium (DMEM) supplemented with 10% fetal bovine serum (FBS) (Sigma-Aldrich) and 1% (v/v) Penicillin-Streptomycin. The medium was replaced every 2 days. When most of the cells became detached from the plates, the cells and culture medium were harvested together, freeze-thawed three times, and centrifuged to give the adenovirus-enriched supernatants.

### Cell culture

A 8505C cell line derived from human thyroid cancer and A549 from lung cancer were provided by the RIKEN BRC. All cells were incubated at 37°C in 5% (v/v) CO2. 8505C cells were grown in MEM (NISSUI) supplemented with 10% (v/v) FBS (SIGMA-ALDRICH), 2 mM L-glutamine (GIBCO) and 1% (v/v) Penicillin-Streptomycin (SIGMA-ALDRICH). A549 cells were in DMEM (NISSUI) supplemented with 10% (v/v) FBS, 2 mM L-glutamine, and 1% (v/v) Penicillin-Streptomycin (SIGMA-ALDRICH). HEK293A cells were in grown in DMEM supplemented with 4.5 mM D-glucose (Wako pure chemicals industries), 10% (v/v) FBS, 2 mM L-glutamine, and 1% (v/v) Penicillin-Streptomycin.

### Immunoblotting

Proteins were resolved by SDS polyacrylamide gel electrophoresis, and then transferred to PVDF membranes (General Electronic). The membranes were blocked for 1 h with blocking buffer containing 3% skim milk and incubated with the diluted primary antibodies, anti-SNX1 (1:200), anti-SNX2 (1:200), anti-SNX5 (1:200), anti-SNX6 (1:200), anti-GAPDH (1:200), anti-FLAG M2 (1:500), followed by secondary antibodies conjugated to peroxidase. The proteins were visualized by LAS-3000 (FUJI FILM) using the Luminate Forte Western HRP Substrate (Millipore).

### Immunofluorescence microscopy

After transfection of the SNX5 cDNA using recombinant adenovirus, the 8505C cells were plated in 8 well chamber slides precoated with 30 μg/mL bovine collagen type I (Wako chemical industry) and then cultured for total 3 days. The cells were fixed in 4% para-formaldehyde for 15 min at room temperature, permeabilized with 0.1% Triton X-100 in PBS for 5 min, blocked by treatment with 3% BSA in PBS for 30 min, and incubated with the primary antibody against FLAG (1:1000) or SNX1 (1:200), SNX2 (1:200), EEA1 (1:200), TGN46 (1:200), CI-M6PR (1:200) for 1 h. The cells were then washed twice with 1% BSA in PBS, incubated with fluorescence-labeled secondary antibody (Alexa Fluor 488, Alexa Fluor 568, 1:1000) at room temperature for 1h and washed twice with 1% BSA in PBS. The intracellular localization of proteins was determined using a Zeiss confocal microscope (LSM710).

### Immunoprecipitation

SNX5 and its mutants fused with the 3×FLAG tag were expressed in 8505C cells for 48 h. The cells were lysed with 1 mL of lysis buffer (0.5% NP-40, 50 mM Tris pH8.0, 150 mM NaCl, 1 mM Na3VO4, 10 mM NaF). After centrifuging the lysate at 4°C for 20 min at 14,000 x g, the cytosol was obtained as the supernatant and was incubated with 10 μL anti-FLAG M2 beads for 4 h at 4°C. The beads were then washed five times with 1 mL of lysis buffer and once with 1 mL of detergent free buffer (50 mM Tris pH8.0, 150 mM NaCl, 1 mM EDTA). The bound proteins were eluted twice by adding 10 μL of 500 μg/mL FLAG-peptide (SIGMA-ALDRICH) in detergent free buffer, separated by SDS-PAGE, and analyzed by western blotting with the indicated antibodies.

For immunoprecipitation with anti-SNX1 antibodies, the above cytosol fractions were incubated with 2 μg of anti-SNX1 antibodies for 1 h and then mixed with 5 μL of Protein G Sepharose beads at 4°C for 4 h. The beads were then washed as described above, and the SNX1 associated proteins were released from the beads by treatment with 10 μL of SDS sample buffer (50 mM Tris-HCl pH6.8, 2% SDS, 5% β-mercaptoethanol, 10% glycerol and 0.05% bromophenol blue), and analyzed by western blotting with the indicated antibodies.

### Circular dichroism (CD) spectroscopy

Maltose binding proteins (MBP) fused recombinant SNX5s were expressed in E. coli using pMAL-cRI expression vector and purified by affinity chromatography with an amylose resin (NEB) according to the manufacture's protocol. The recombinant purified proteins were dissolved in 20 mM Tris pH7.4, 200 mM NaCl at a concentration of 0.1 mg/mL. CD spectra were recorded on a J-720WI spectrophotometer (Jasco GmbH) at a 0.01/cm optical path, a 0.5 nm interval, a 1.0 nm bandwidth and a 50 nm/min scanning speed. The spectra were recorded five times followed by averaging and background subtraction. The HT voltage was below 700 V over the entire range of recording (200–250 nm).

### Fluid-phase uptake assay

2×10^5^ cells of 8505C were plated in 8 well chamber slide precoated with 30 μg/mL bovine collagen type I (Wako chemical industry). After an overnight culture, the cells were incubated in Opti-MEM (GIBCO) with 500 μg/mL FITC-conjugated 70 k dextran and 100 ng/mL of EGF at 37°C for 15 min. Cells were washed twice with cold PBS and scanned by a DMI6000B scanner (Leica). The number of macropinosomes was counted visually for each cell in which FITC-dextran was taken up.

### siRNA treatment

siRNA targeting SNX5 and SNX6 were purchased from Invitrogen. Their target sequences were as follows: SNX5 (5’-CCCTCATTGACTATGAGAACTCAAA-3’) and SNX6 (5’-AGTGCTGCAGATGATTACAATAGAA-3’). 8505C cells were transfected with 20 nM siRNA according to the manufacture’s protocol using RNAiMAX (Invitrogen). Knockdown efficiency was determined by immunoblotting. For the siRNA rescue assay, seven silent mutations were introduced to the siRNA targeting sequence as follows, 5’-CTCTGATCGATTACGAGAATTCCAA -3’.

## Results

### Identification of phosphorylation sites on SNX5

In previous studies, we observed dramatic morphological changes in Colo201 cells upon treatment with 40 nM staurosporine, a protein kinase inhibitor [25]. To investigate the cause of this phenomenon, lysates from staurosporine treated and untreated Colo201 cells were analyzed by a two-dimensional differential in-gel electrophoresis technology (2D-DIGE) system and the results compared. Protein spots were imaged by means of a Typhoon 9400 instrument and about 4,000 separate spots were detected (S1 Fig). Twentytwo spots were excised from the 2D-DIGE gel and 10 spots were identified, and finally 3 proteins were discovered to have had their isoelectric points changed, as evidenced by LC-MS/MS analysis. One of them was SNX5. Although there are some reports that SNX5 contains phosphorylation sites [26–27], their importance has not been elucidated. Thus, we attempted to clarify the significance of the phosphorylation of SNX5.

**Fig 1:**
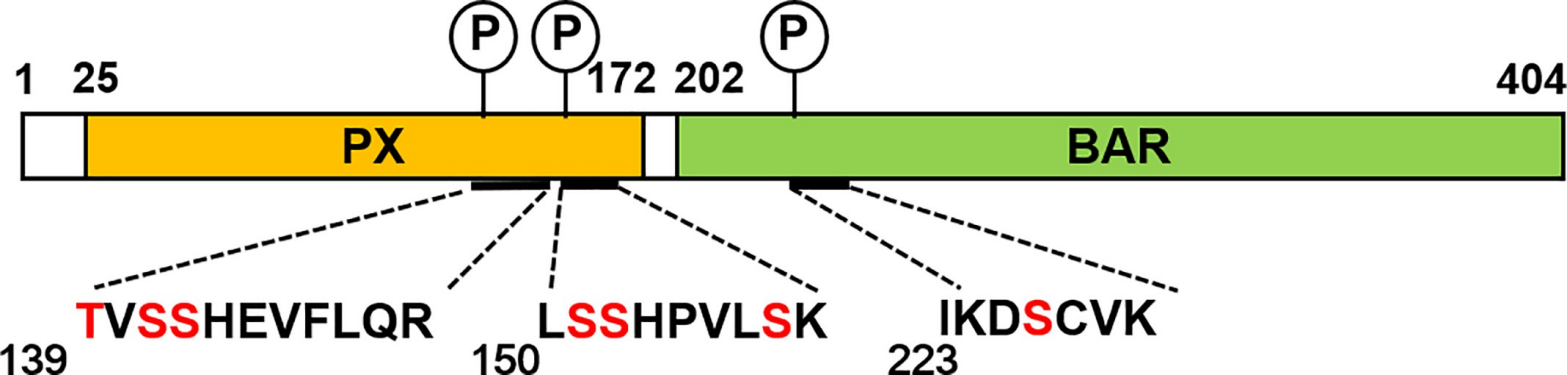
SNX5 phosphorylation sites. **A schematic diagram of SNX5.** Three tryptic peptides containing one phosphorylation site each were detected. Candidate phosphorylation residues are shown in red. PX; phox homology domain, BAR; Bin-Amphiphysin-Rvs domain.

First, FLAG-tagged SNX5 was expressed in A549 cells using an adenovirus protein expression system and immunoprecipitated with anti-FLAG beads. The eluate by FLAG-peptide was examined by 2D-PAGE and three spots were detected by silver staining (S2 Fig). Each spot was excised, treated with trypsin, and the digested peptides were analyzed by ESI-MS/MS (S3 Fig). Three phosphorylated peptides were identified (Fig 1). The tryptic peptide from I223 to K229 in the BAR domain contains S226 as a candidate phosphorylation site, and both peptides from T139 to R149 and L150 to K158 in the PX domain had three candidate phosphorylation sites, respectively.

### Effect of phosphorylation on heterodimerization

Because all of these novel phosphorylation sites were located in the functional domain of SNX5, we were prompted to ask whether the phosphorylation of these sites had any effect on their function. To address this question, we initially prepared six FLAG-tagged SNX5 mutants that mimic phosphorylated or non-phosphorylated SNX5. Since three phosphorylation candidate sites were present within the tryptic peptide from T139 to R149, as shown in Fig 1, we modified all three residues to glutamic acid or alanine residues. The mutants were as follows: the mimic phosphorylated mutants were TSS-EEE, T139E/S141E/S142E; SSS-EEE, S151E/S152E/S157E; S226E, and the mimic non-phosphorylated mutants were TSS-AAA, T139A/S141A/S142A; SSS-AAA, S151A/S152A/S157A; S226A. An adenovirus expression system was used to express the mutant protein in 8505C derived human thyroid cancer cells.

**Fig 2:**
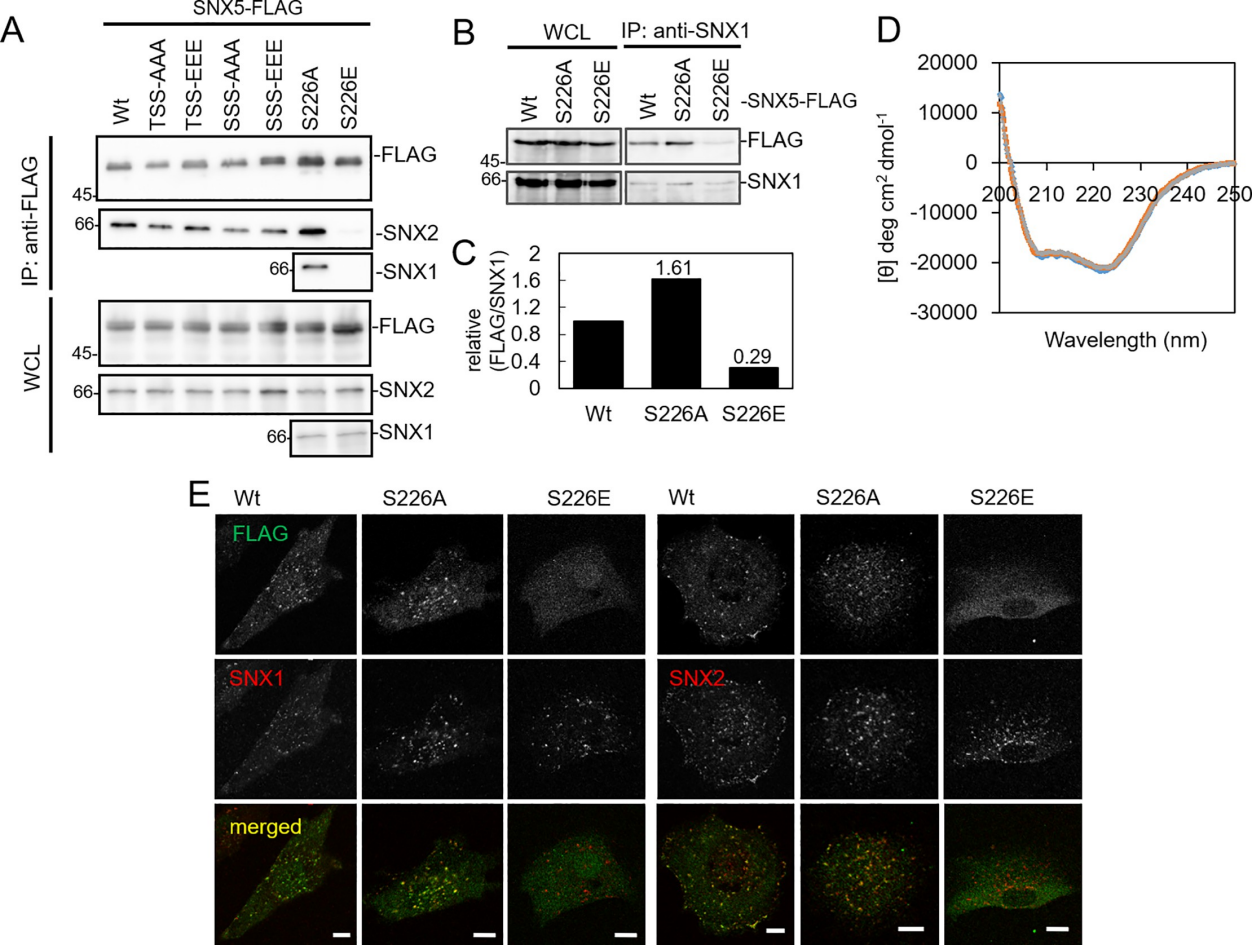
The influence of S226 phosphorylation on the interaction between SNX5 and SNX1 or SNX2. (A) FLAG pull-down assay was performed on lysates of 8505C cells which were expressed SNX5-FLAG wild type (Wt), or a mutant, T139A/S141A/S142A (TSS-AAA)), T139E/S141E/S142E (TSS-EEE), S151A/S152A/S157A (SSS-AAA), S151E/S152E/S157E (SSS-EEE), S226A, S226E, by an adenovirus expression system. FLAG fused proteins (SNX5), SNX1 and SNX2 were detected by immunoblotting with anti-FLAG, anti-SNX1, anti-SNX2 antibodies. (B) Immunoprecipitation using anti-SNX1 antibody was performed on lysates of 8505C cells which were expressed FLAG-tagged SNX5 wild type, S226A, and S226E by adenovirus expression system. FLAG fused proteins and SNX1 were detected by immunoblotting with anti-FLAG and anti-SNX1 antibodies, respectively. (C) The band intensity of (B) were measured and quantified. (D) CD spectra of MBP fused SNX5 wild type (blue), S226A (red), S226E (green). (E) Immunocytochemical staining of 8505C cells which were expressed by SNX5-FLAG wild type, S226A, and S226E. The cells were stained with anti-FLAG (green), anti-SNX1 (red) and anti-SNX2 (red) antibodies and observed using confocal microscopy. Scale bars: 10 μm.

SNX5 is known to form a hetero-dimer with SNX1 or SNX2 in cells [13]. To examine the effect of SNX5 phosphorylation on interaction with SNX1 and SNX2, mimic phosphorylated and non-phosphorylated SNX5s were expressed in 8505C cells, and an immunoprecipitation assay was carried out using anti-FLAG beads. As shown in Fig 2A, the wild type and all the mimic phosphorylated and non-phosphorylated SNX5s expect S226E mutant interacted to SNX2. In addition, S226E did not interact with SNX1. Next, we carried out a co-immunoprecipitation with SNX5 expressing cells using an SNX1 antibody. The binding of SNX1 to the SNX5 S226E mutant was decreased whereas binding to the S226A mutant was similar to that seen with wild type SNX5 (Fig 2B and 2C). In order to look for potential structural changes in the S226E mutant, the maltose binding protein (MBP) fused to SNX5 wild type, S226A and S226E proteins were expressed in E. coli, purified by affinity chromatography, and analyzed by circular dichroism (CD) (Fig 2D). All CD spectra were very similar to each other, indicating that the mutations did not lead to detectable changes in the secondary structure or the folding properties of the SNX5 mutants.

The colocalization of SNX1 or SNX2 with SNX5 mutants in the cells were also examined. FLAG-tagged wild type, S226A and S226E were expressed in 8505C cells and their colocalization examined by confocal microscopy. As shown in Fig 2E, the SNX5 wild type and S226A colocalized with SNX1 and SNX2 at cytoplasmic puncta. In contrast, S226E had a diffuse appearance in the cytosol and did not colocalize with either SNX1 or SNX2.

### Effect of S226 phosphorylation on SNX5 localization in cells

**Fig 3:**
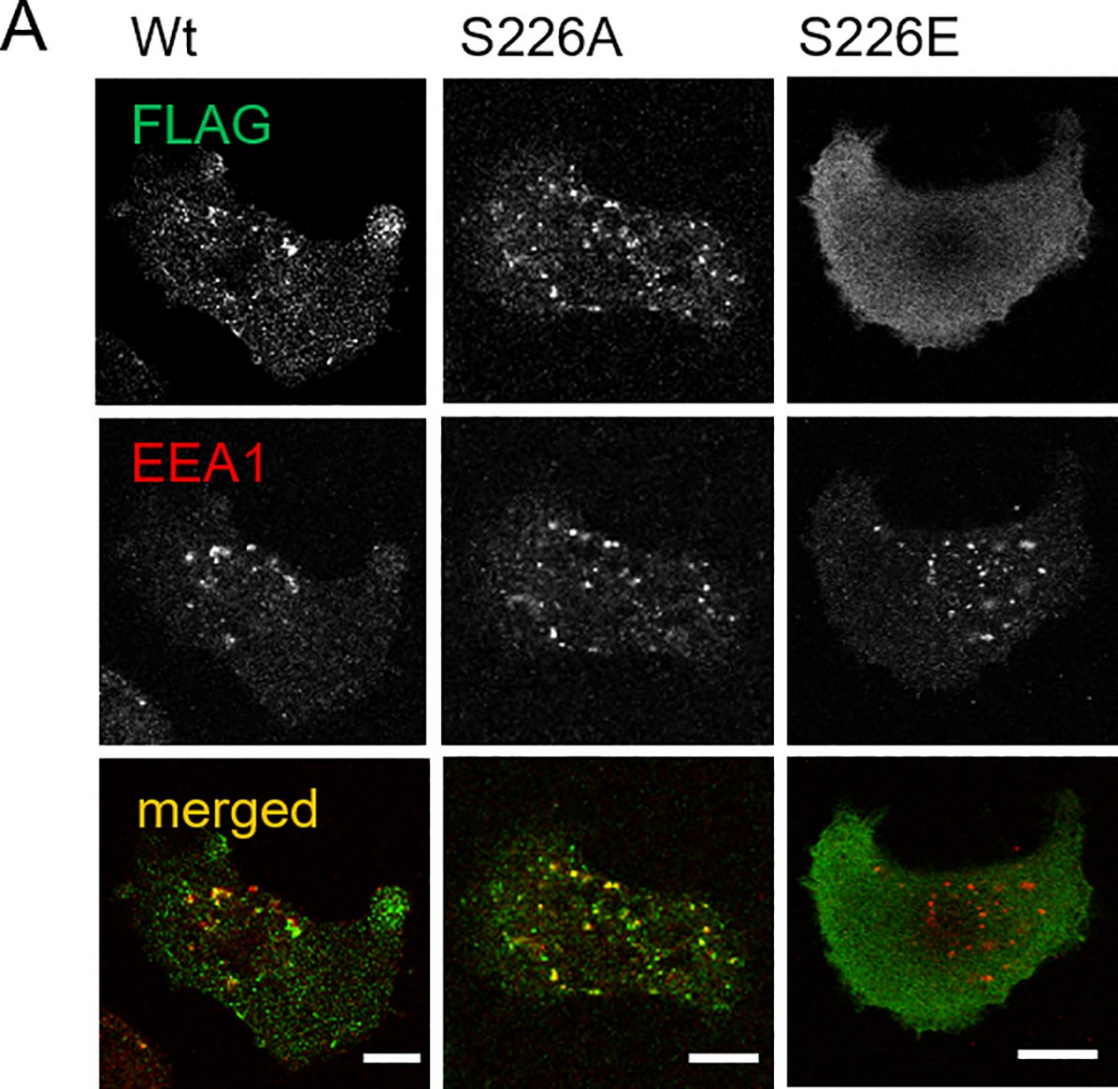
Changes in the localization of SNX5 S226 mutants. (A)FLAG-tagged SNX5 wild type (Wt), S226A, and S226E were expressed in 8505C cells using an adenovirus expression system. The cells were stained with anti-FLAG (green) and anti-EEA1 (red) antibodies and observed by confocal microscopy. Scale bars indicate 10 μm.

Previous studies indicated that SNX5 is localized in the endosomal compartment [28]. To test whether SNX5 localization was influenced by S226 phosphorylation, we observed the localization of SNX5 in wild type and S226 mutants in 8505C cells. As shown in Fig 3A, SNX5 S226A and wild type colocalized with the endosomal marker EEA1 whereas negligible amounts of the S226E mutant colocalized with this marker.

### Influence of S226 phosphorylation on CI-M6PR retrograde transport

**Fig 4:**
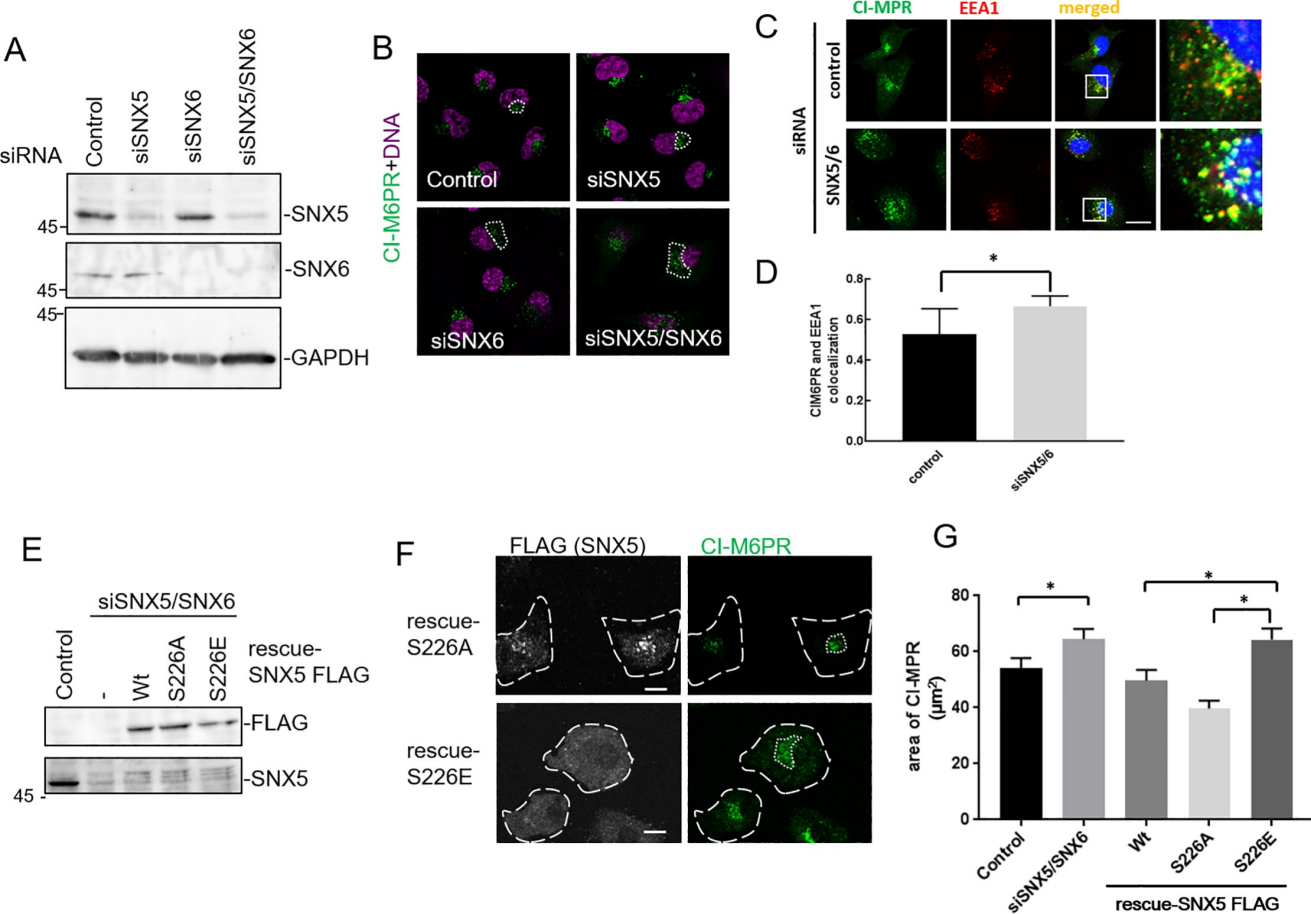
Effect of SNX5 S226 phosphorylation on the retrograde transport of CI-M6PR. (A-D) 8505C cells were treated with control, SNX5, SNX6, SNX5/SNX6 siRNA for 72 h. (A) Cell lysates were immunoblotted with anti-SNX5, anti-SNX6, and anti-GAPDH antibodies. (B) The cells were stained with anti-CI-M6PR (green) and DAPI (magenta) and observed by confocal microscopy. The dotted lines indicate the diffuse area of CI-M6PR, which was decided after thresholding by image J. (C) The cells were stained with anti-CI-M6PR (green), anti-EEA1 (red) antibodies and DAPI (blue). The boxed region is magnified on the right. Scale bars indicate 10 μm. (D) The colocalization of CI-M6PR with EEA1 was quantified. The results shown are mean±SEM of 30 cells from three independent experiments. Asterisk indicates; p<0.05 by Student’s t-test. (E-G) 8505C cells were treated with control or SNX5/SNX6 siRNA for 48 h and then infected by adenovirus bearing rescue-FLAG-tagged SNX5 S226A or S226E. (E) After 24 h, cell lysates were immunoblotted with anti- FLAG and anti-SNX5 antibody. SNX5 derived from endogenous and transfection were indicated. (F) The cells were immunostained with anti-FLAG (gray) and anti-CI-M6PR (green) antibodies. The long-dashed lines express shape of the cells and dotted lines indicate the diffuse area of CI-M6PR. Scale bars indicate 10 μm. (G) The area of CI-M6PR in each cell are plotted. The results shown are mean±SEM of 127 cells from three independent experiments. Asterisk indicates; p<0.05 by Student’s t-test.

SNX-BAR dimers (SNX1/2 and SNX5/6) are reported to be involved in tubulation from endosome in retrograde transport [18–20]. To assess this, we examined the influence of S226E on CI-M6PR retrograde transport from endosomes to TGN using an siRNA protein knock-down system. Since SNX6 is a homolog of SNX5 and performs a similar function as a component of the retromer [21], we generated SNX5, SNX6 and SNX5/SNX6 double knock-down cells (Fig 4A). As shown in Fig 4B, in SNX6 and SNX5/SNX6 knockdown cells, the region of localization of CI-M6PR was comparatively diffuse in the cells. In addition, the amount of CI-M6PR that was colocalized with early endosomes appeared to be increased by comparison of Pearson’s correlation (Fig 4C and 4D). Although no expansion of the CI-M6PR localization area was observed in SNX5 knockdown cells, we concluded that SNX6, a homolog of SNX5, was preferentially recruited into SNX dimerization in 8505C cells.

We next expressed rescue-SNX5 S226A or S226E mutants in SNX5/SNX6 double knockdown cells by adenovirus infection methods (Fig 4E). As shown in Fig 4F, the CI-M6PR localization of SNX5 S226A rescued cells was returned to that of control cells, but this was not the case for the S226E rescued cells. Quantification analysis shows the area of CI-M6PR in each cell (Fig 4G). These results suggest that phosphorylation at the S226 residue causes the function of SNX5 to be lost.

### Effect of S226 phosphorylation on macropinocytosis

Kerr et al. reported that the association of SNX5 with tubular extensions of macropinosomes and SNX5-positive tubules play a role in macropinosome to early endosome trafficking [10]. It was also reported that SNX5 functioned as a modulator of macropinocytosis and functioned independently from SNX1 in mouse macrophages [29–30]. We then examined whether the phosphorylation of SNX5 S226 affected its function as a regulator of macropinocytosis.

**Fig 5:**
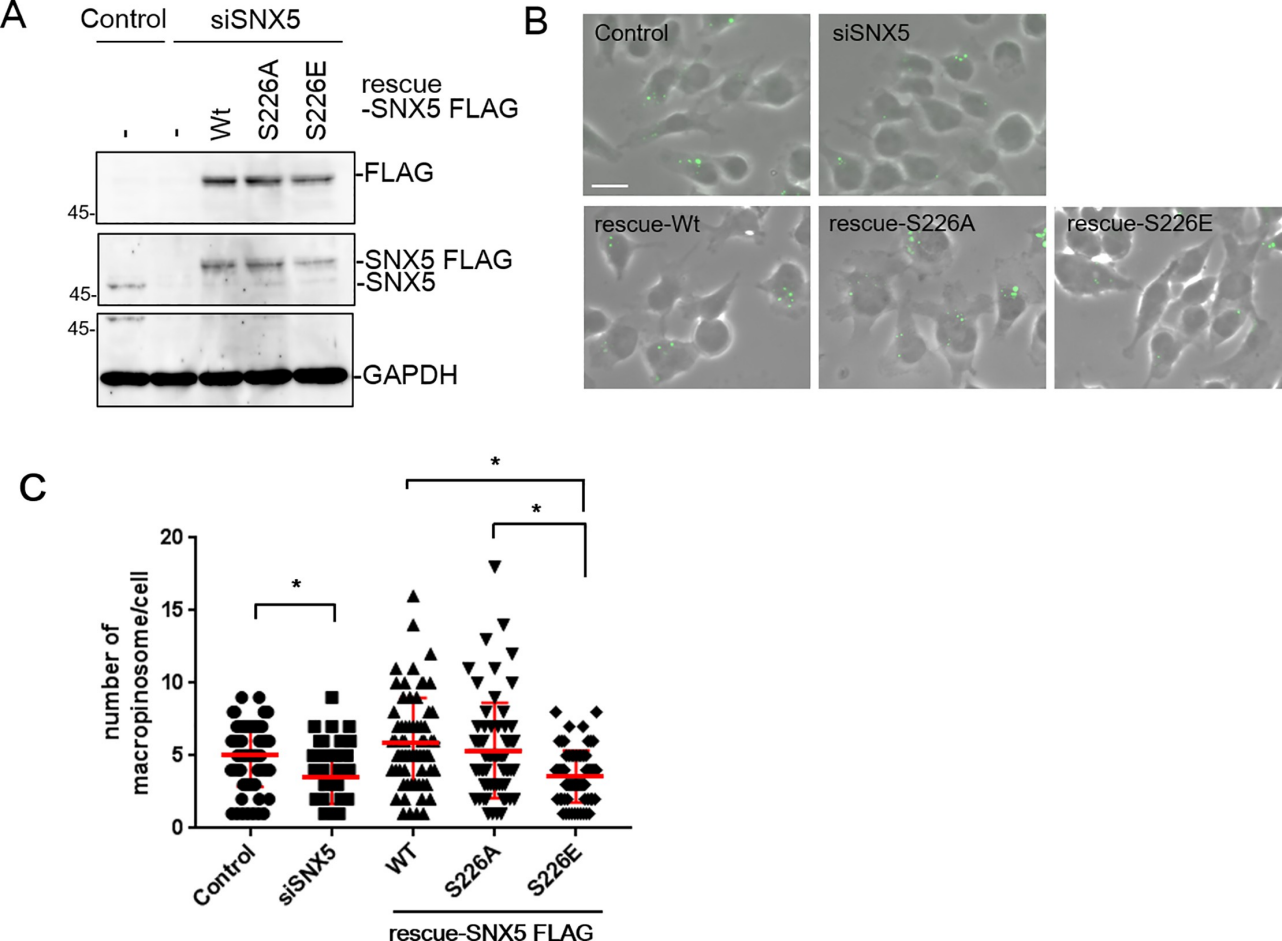
Effect of SNX5 S226 phosphorylation on macropinocytosis. 8505C cells were treated with control or SNX5 siRNA for 48 h and rescue-SNX5-FLAG WT, S226A and S226E were then expressed for 24 h by adenovirus infection. (A) Cell lysates were immunoblotted with anti-FLAG, anti-SNX5 and anti-GAPDH antibodies. (B) After the rescue proteins had been expressed, each of the cell preparations were incubated with 500 μg/mL of FITC-conjugated 70 kDa dextran in the presence of 100 ng/mL EGF for 15 min. (C) The number of vesicles which containing FITC-dextran were counted in each cell. Data for 68 cells from three independent experiments are represented as the mean ±SEM. Asterisk indicates; p<0.001 by Student’s t-test.

We initially confirmed that SNX5 also regulates macropinocytosis with EGF stimulation in 8505C cells. The cells treated with control or SNX5 siRNA were incubated with fluorescein isothiocynate conjugated 70 kDa dextran (FITC-dextran) in the presence of 100 nM EGF for 15 min at 37°C to induce macropinocytosis. As shown in Fig 5A and 5B, some of the particles in the siSNX5 treated cells containing FITC-dextran macropinosomes, were decreased, as compared with control cells. Although the depletion of SNX6 also decreased macropinosomes, the effect is less than SNX5 (result not shown). Moreover, the number of macropinosomes were restored by the expression of siRNA-resistant FLAG-tagged SNX5 wild type and S226A (rescue-Wt and rescue-S226A) in SNX5 knockdown cells. On the other hand, expression of the rescue S226E mutant did not restore the capacity for macropinocytosis (Fig 5C). These results show that the phosphorylation of SNX5 S226 also regulates macropinocytosis as well as the retrograde transport of CI-M6PR.

## Discussion

**Fig 6:**
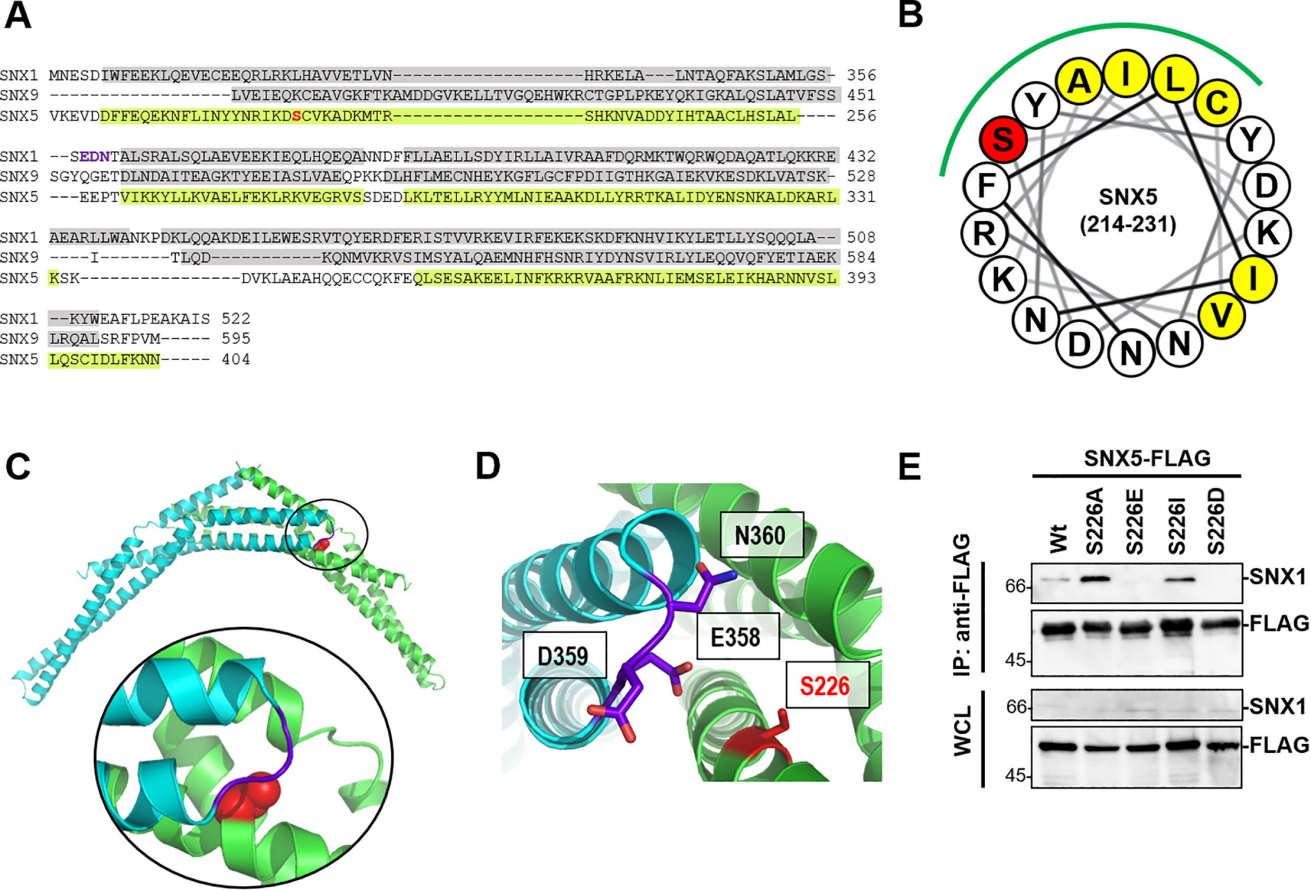
SNX5 S226 localize in the hydrophobic region of α-helix on BAR domain. (A) Alignment of the BAR domain of three SNX family members. The α-helices (gray) of SNX1 and SNX9 are indicated based on the SNX1 crystal structure and the SNX9 structure. The α-helices of SNX5 (light green) are predicted by PSI-PRED. (B) Cartoon of the arrangement of the 18-residue around S226 (red). Green line shows hydrophobic region in which hydrophobic residue is assembled. (C) The model of the heterodimer of SNX1-BAR and SNX5-BAR. SNX1-BAR and SNX5-BAR are shown in cyan and green, respectively. S226 is shown as red space filling sphere. Around S226 is extended in circle. (D) Close-up view of S226 in the interface of the heterodimer of SNX1-BAR and SNX5-BAR. The charged residues in SNX1-BAR within 10Å of S226 and S226 are shown in stick format. Images (C) and (D) were produced using PyMOL softwere. (E) FLAG pull-down assays were performed on lysates of HEK293T cells expressing SNX5 Wt, S226A, S226E, S226I and S226D by lipofection. The FLAG fusion protein and SNX1 were detected by immunoblotting with anti-FLAG, anti-SNX1 antibodies.

The phosphorylation of S226 of SNX5 is a key regulatory step for interactions between SNX5:SNX1 and/or SNX5:SNX2 as indicated by our experiments (Figs 2 and 3). The structural mechanism for the interaction between these proteins is intriguing, but there is no report concerning the three-dimensional structure of the BAR domain of SNX5, where S226 is located. A clue for this could be found by a sequence alignment among the BAR domains of SNX1 (21%), SNX9 (44% identity), and SNX5. The structures of SNX1-BAR (PDB ID, 4FZS) and SNX9-BAR (3DYU) have been reported [13,31], and the findings indicate that both structures are composed of multiple α-helicies, and the alignment showed that S226 of SNX5 is also potentially located in an α-helical structure (Fig 6A). In the homodimer structure of SNX1, no significant hydrogen bonding was observed between the two SNX1 monomers, and a hydrophobic environment has been proposed to be the most important for the dimer formation. A topology diagram of the α-helix in the range of residues 214–231 of SNX5 suggested that S226 is present in a hydrophobic surface (indicated in green in Fig 6B) which is likely to play an important role in the heterodimerization of SNX1:SNX5.

To examine the interface of the SNX1:SNX5 heterodimer further, a homology model of SNX5 was constructed based on the SNX1-BAR structure using the Swiss-Model server. Modelling of the SNX5-BAR has also been reported previously, and the model obtained here appears to be similar, since E280 was located close to E383, as described in a previous paper [13]. A model of the SNX1:SNX5 heterodimer was then constructed by superimposing the obtained model of SNX5 with the structure of the SNX1-BAR homodimer (Fig 6C). In the resulting model, S226 faces into the SNX1:SNX5 heterodimer interface, as expected, and S226 is predicted to be present close to E358 in SNX1 (Fig 6D). A study of the heterodimer formation of SNX1:SNX8 [27] indicated that the interaction between a charged residue, SNX1-R337, and SNX8-Q310 restrict the dimerization process. Therefore, it is likely that the interaction between SNX5-S226 and SNX1-E358 are related to dimer formation of SNX1:SNX5. To confirm that the charge in phosphorylation prevents SNX1:SNX5 heterodimerization, we mutated it to hydrophobic residues, namely, alanine (S226A) and isoleucine (S226I), or charged residues, glutamic acid (S226E) and aspartic acid (S226D) and tested their ability to undergo dimerization through immunoprecipitation in HEK293T cells. The replacement of S226 with charged residues unlike hydrophobic residues prevented interactions between SNX1 and SNX5 (Fig 6E). These results strongly indicate that the charge of the phospho-S226 unit in the SNX5-BAR effects heterodimerization.

The PX domain is involved in diverse functions such as cell signaling and vesicular trafficking [11,32], and is important for binding to phosphoinositides (PtdIns). The BAR domain has been reported to be involved in a variety of cellular functions, including phagocytosis, endocytosis, apoptosis and cell division [33]. Proteins that contain the PX and the BAR domain, the SNX protein family, play extremely important roles in the regulation of tubular-based trafficking events among the endocytic network [32]. In the case of A549 cells under normal culture conditions, the abundance of phosphorylated SNX5 at S226 is low (S3 Fig). It would be very interesting to have information on the relationship between the metabolic status of the cell and the increased phosphorylation status of S226. To address this, we are in the process of preparing a monoclonal antibody against phosphorylated S226. On the other hand, other phosphorylation sites that we identified in the PX domain were found to not be important for the formation of dimers. Recently, Lenoir et al. showed phosphorylation of the critical conserved serine residues in SNX proteins (excluding SNX5 and SNX6) in phosphoinositide binding pocket on PX domain regulated SNX membrane targeting [34]. However, the function of phosphorylation in the SNX5 PX domain is still unclear. How membrane trafficking is controlled by multiple phosphorylation and what kind of kinase functions that could clarify these tasks will be our next themes.

## Supporting information

S1 Fig 2D-DIGE analysis. (A) 2D-DIGE images of Colo201 cells treated with (green) and without (red) 100 nM staurosporine. 2D-DIGE analysis of the differentially expressed spots between staurosporine treated cells and untreated cells. Immobiline DryStrip 24 cm gel (Amersham), pI range 4–7, was used for the first dimension and a 10% concentration gel was used for the second dimension. (B) Enlarged picture of a framed box in (A). Two arrows show spots of SNX5. (C) The log standardized abundance for spot a in (B). The signal intensities for each condition are connected by a line. Blue line shows an average line. The signal intensity was significantly decreased in the case of the staurosporine treated cells. Student’s t-test; p<0.00025.
(TIF)

S2 Fig Purification of FLAG-tagged SNX5. **Affinity purified FLAG-tagged SNX5 was subjected to 2D-PAGE.** (A) Silver stained 2D-PAGE gel. ReadyStrip 7 cm IPG (Bio-Rad), pI range 5.5–6.7, was used for the first dimension and a 10% concentration gel was used for the second dimension. (B) Enlarged picture of the framed box in (A). Three spots which were identified as being SNX5 by MS spectroscopy and immunoblotting were collected for the next step.
(TIF)

S3 Fig Nano-LC/ESI-MS/MS of the tryptic digest of spot L in S2 Fig. **Mass chromatograms were obtained for m/z 425.2 and 465.2** (A), m/z 484.3 and 524.2 (B), and m/z 651.8 and 691.8 (C), each pair of which was identified as the peptide and its phosphorylated form of I^223^KDSCVK^229^ (m/z_theo._ 425.2(+2)), L^150^SSHPVLSK^158^ (m/z_theo._ 484.3(+2)), and T^139^VSSHEVFLQR^149^ (m/z_theo._ 651.8(+2)), respectively. The phosphorylated peptides were eluted at 8.7 (A), 24.4 (B), and 29.1 min (C), and observed to be higher in mass by 80 Da compared to the non-phosphorylated materials obtained at 6.3 (A), 21.2 (B), and 26.6 min (C), respectively. The spot L contained both phosphorylated and unphosphorylated peptides. Therefore, it seems that the spot L was not derived from a single phosphorylated form. Similarly, multiple phosphorylated forms were found for spot K and spot M as well.
(TIF)

S1 File Supplemental experimental procedure. (DOCX)

S2 File Supporting data set. (XLSX)
