# Supplementary figures and images for: The phosphorylation of sorting nexin 5 at serine 226 regulates retrograde transport and macropinocytosis
Source: PLoS One. 2018 Nov 12;13(11):e0207205. doi: 10.1371/journal.pone.0207205 (PMC6231649; doi:10.1371/journal.pone.0207205)

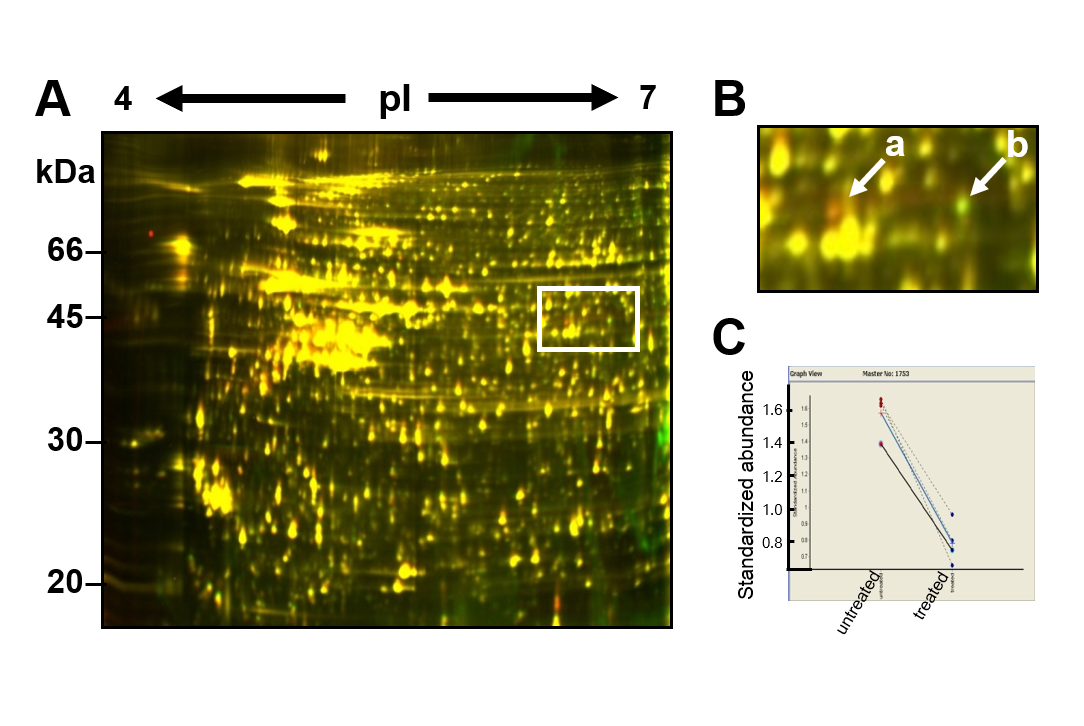

Supplement: S1 Fig — (A) 2D-DIGE images of Colo201 cells treated with (green) and without (red) 100 nM staurosporine. 2D-DIGE analysis of the differentially expressed spots between staurosporine treated cells and untreated cells. Immobiline DryStrip 24 cm gel (Amersham), pI range 4–7, was used for the first dimension and a 10% concentration gel was used for the second dimension. (B) Enlarged picture of a framed box in (A). Two arrows show spots of SNX5. (C) The log standardized abundance for spot a in (B). The signal intensities for each condition are connected by a line. Blue line shows an average line. The signal intensity was significantly decreased in the case of the staurosporine treated cells. Student’s t-test; p<0.00025. (TIF) [file pone.0207205.s001.tif]

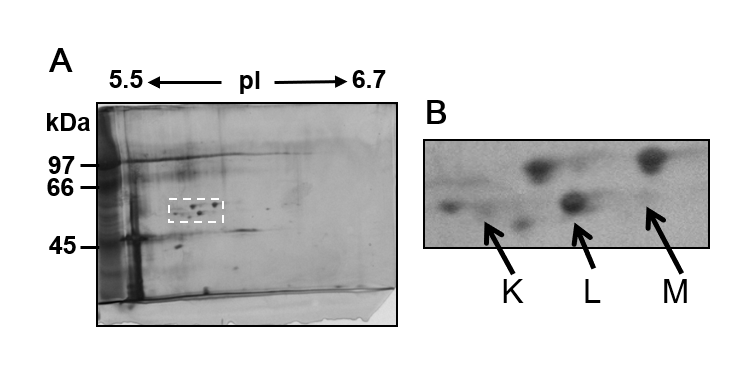

Supplement: S2 Fig — Affinity purified FLAG-tagged SNX5 was subjected to 2D-PAGE. (A) Silver stained 2D-PAGE gel. ReadyStrip 7 cm IPG (Bio-Rad), pI range 5.5–6.7, was used for the first dimension and a 10% concentration gel was used for the second dimension. (B) Enlarged picture of the framed box in (A). Three spots which were identified as being SNX5 by MS spectroscopy and immunoblotting were collected for the next step. (TIF) [file pone.0207205.s002.tif]

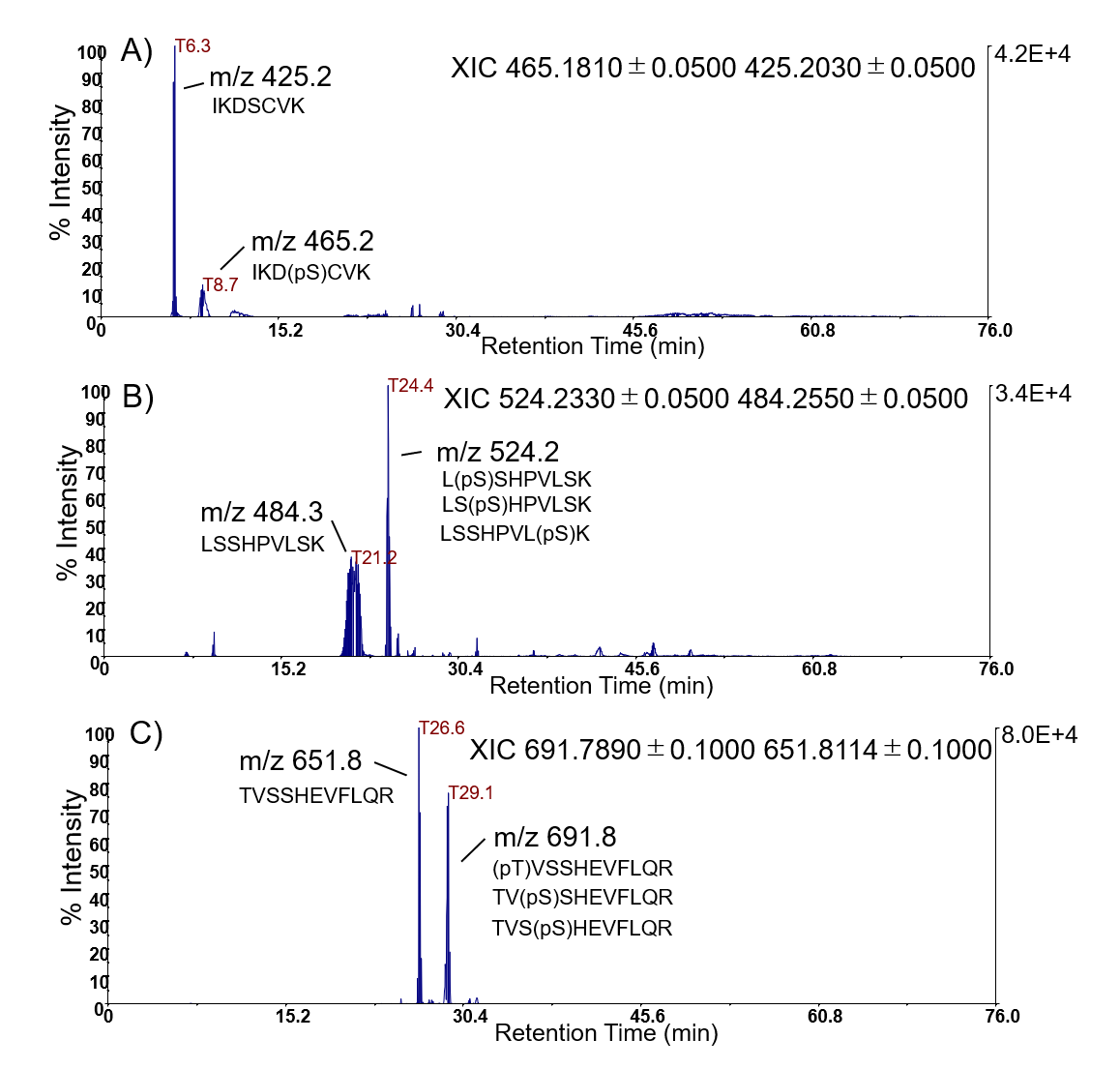

Supplement: S3 Fig — Mass chromatograms were obtained for m/z 425.2 and 465.2 (A), m/z 484.3 and 524.2 (B), and m/z 651.8 and 691.8 (C), each pair of which was identified as the peptide and its phosphorylated form of I223KDSCVK229 (m/ztheo. 425.2(+2)), L150SSHPVLSK158 (m/ztheo. 484.3(+2)), and T139VSSHEVFLQR149 (m/ztheo. 651.8(+2)), respectively. The phosphorylated peptides were eluted at 8.7 (A), 24.4 (B), and 29.1 min (C), and observed to be higher in mass by 80 Da compared to the non-phosphorylated materials obtained at 6.3 (A), 21.2 (B), and 26.6 min (C), respectively. The spot L contained both phosphorylated and unphosphorylated peptides. Therefore, it seems that the spot L was not derived from a single phosphorylated form. Similarly, multiple phosphorylated forms were found for spot K and spot M as well. (TIF) [file pone.0207205.s003.tif]
