# Supplementary material for: The phosphorylation of sorting nexin 5 at serine 226 regulates retrograde transport and macropinocytosis
Source: PLoS One. 2018 Nov 12;13(11):e0207205. doi: 10.1371/journal.pone.0207205 (PMC6231649; doi:10.1371/journal.pone.0207205)
Supplement: S1 File — (DOCX) [file pone.0207205.s004.docx]

**Supplemental Experimental procedures**

**2D-DIGE analysis**

Two-dimensional differential in-gel electrophoresis technology (2D-DIGE) analysis (Amersham) was carried out according to the manufacture's methods as follows. Whole cell lysates, which were produced from Colo201 cells treated with 100 nM staurosporine (Kyowa Co. Japan) for 1 h and untreated control cells, were prepared with lysis buffer (7 M urea, 2 M thiourea, 4% (w/v) CHAPS, 2 mM Na_3_VO_4_, 10 mM NaF, 30 mM Tris-HCl pH8.5, and protease inhibitor cocktail (Sigma-Aldrich). After centifugation, the supernatants were concentrated to 50 μg protein/ 8 μL. Fifty microgram samples of protein were labeled by 400 pmol Cydye and incubated on ice for 30 min in the dark. Staurosporine treated samples obtained from 4 independent experiments and 4 untreated samples from 4 experiments were labeled separately with either Cy3 or Cy5. On the other hand, to prepare the internal standard, 4.2 μL from each of 8 samples were mixed and labeled with Cy2. The different Cydye labeled staurosporine treated samples and untreated sample and a portion of a Cy2 labeled internal standard were mixed and added to the same quantity of 2×sample buffer (7 M urea, 2 M thiourea, 4% w/v CHAPS, 130 mM DTT, 2% (v/v) Pharmalyte), and finally added 390 μL of rehydration buffer (7 M urea, 2 M thiourea, 4% w/v CHAPS, 13 mM DTT, 1% (v/v) Pharmalyte).

The first dimensional separations were performed using Immobiline DryStrip gel (Amersham) by applying combined samples to 24 cm pH 4-7 strips. After electrophoresis, the strip was incubated in 0.1 M Tris buffer pH8.0 containing 0.5% DTT, 6M urea, 30% (v/v) glycerol and 2% (w/v) SDS for 15 min, and subsequently incubated with the above buffer containing 4.5% (w/v) iodoacetamide for 15 min. SDS polyacrylamide gel electrophoresis for the second dimension were carried out using a 10% concentration gel. After electrophoresis the gel was scanned by means of a Typhoon9400 image scanner (Amersham). The scanning conditions were as follows, Cy2 laser: 488 nm, emission filter: 520BP40, Cy3 laser: 532 nm, emission filter: 540BP30, Cy5 laser: 633 nm, emission filter: 670BP30. Each image was analyzed with statistics analysis software DeCyder (Amersham).

**Identification of SNX5**

Twenty-two spots were excised from the 2D-DIGE gel and 10 spots were ultimately identified and 3 proteins were discovered as having their isoelectric point changed by LC-MS/MS analysis. The excised gel pieces were destained twice with 100 mM ammonium bicarbonate, 50% acetonitrile at 37°C for 45min. The dried gels following treatment of 100% acetonitrile were soaked in a trypsin solution (20 µg trypsin (Promega) in 40 mM ammonium bicarbonate, 10% acetonitrile) at 37°C for overnight. Then 50 µL of water was added and peptides were extracted with vigorous stirring for 60 min. Next, 50 µL of 50% acetonitrile in 5% TFA was added and the extraction repeated twice. The digests were evaporated and dissolved in 20 µL of 2% acetonitrile in 0.1% TFA. The samples were applied to LCQ-DECA XP Plus Mass Spectrometry (Thermo Electron). Searches of LC-MS/MS data were done using Mascot database (Matrix Science).

**Sample preparation and purification for mass spectrometry**

We constructed adenovirus bearing recombinant SNX5, the 3xFLAG was fused in the C-terminal of SNX5 and expressed in A549 cells. FLAG-tagged SNX5 expressed cells were suspended in 400 μL of lysis buffer (0.5 % NP-40, 50 mM Tris pH7.4, 150 mM NaCl, 1 mM EDTA, 1% Triton X-100, 1 mM Na_3_VO_4_, 10 mM NaF) and sonicated with BIO-RUPTOR (COSMO BIO). After removing cell debris by centrifugation, the supernatants were applied to Anti-FLAG M2 Agarose beads (Sigma-Aldrich), and FLAG-tagged SNX5 were eluted with a FLAG-peptide (Sigma-Aldrich). The desalted samples were then separated by 2D-PAGE. ReadyStrip IPG pI range 5.5-6.7, 7 cm, Strip (Bio-Rad) was used for the first dimension. After completion of the electrophoresis, the strip was soaked in 50 mM Tris buffer pH8.8 containing 1% DTT, 6M urea, 30% glycerol and 2% (w/v) SDS for 30 min, and subsequently incubated with the above buffer containing 2.5% (w/v) iodoacetamide for 30 min. It was then subjected to a second SDS-electrophoresis followed by silver staining.

FLAG-tagged SNX5 spots excised from 2D-PAGE gel were destained twice with 20 mM EDTA, 50mM ammonium bicarbonate, 30% acetonitrile for 5 min at room temperature, and washed twice by water. The gels were vortexed with a 1:1 mixed solution of 30 mM potassium ferricyanide and 100 mM sodium thiosulfate for 10 min at room temperature and washed twice by water. The dried gels were treated with 50 nM trypsin (Promega) in 10 mM ammonium bicarbonate and 10% acetonitrile for overnight at 37°C. The tryptic peptides were extracted from the gels twice with 100 µL of 0.1% trifluoroacetic acid in 10% acetonitrile.

**Nano-flow liquid chromatography/electrospray ionization (ESI) mass spectrometry**

The above digests were applied to a PicoFrit C18 column (0.075 mm × 100 mm, Inertsil 3 μm particle size; NewObjective; Woburn, MA), and the eluates were then continuously analyzed by a linear ion-trap/time-of-flight mass spectrometer equipped with a nano-flow electrospray ion source (Hitachi NanoFrontier LD; Hitachi High-Technology, Japan) as previously described [35].

35. Kimura M, Kose S, Okumura N, Imai K, Furuta M, Sakiyama N, et al. (2013) Identification of cargo proteins specific for the nucleocytoplasmic transport carrier transportin by combination of an in vitro transport system and stable isotope labeling by amino acids in cell culture (SILAC)-based quantitative proteomics. Molecular & cellular proteomics : MCP 12: 145-157.
